# Ill Effects of Smoking: Baseline Knowledge among School Children and Implementation of the “AntE Tobacco” Project

**DOI:** 10.1155/2011/584589

**Published:** 2011-03-14

**Authors:** Salim Surani, Raghu Reddy, Amy E. Houlihan, Brenda Parrish, Gina L. Evans-Hudnall, Kalpalatha Guntupalli

**Affiliations:** ^1^Pulmonary, Critical Care and Sleep Medicine, Department of Medicine, Baylor College of Medicine Houston and Texas A&M University, 613 Elizabeth Street, Suite 813, Corpus Christi, TX 78404, USA; ^2^Department of Medicine, University of Arkansas, 4301 West Markhan Street, Suite 555, Little Rock, AR 72205, USA; ^3^Department of Psychology, Texas A&M University, 6300 Ocean Drive, Unit 5827, Corpus Christi, TX 78412, USA; ^4^Pulmonary Associates of Corpus Christi, 613 Elizabeth Street, Suite 813, Corpus Christi, TX 78404, USA; ^5^Department of Medicine-Chronic Disease, Baylor College of Medicine, One Baylor Plaza, Houston, TX 77030, USA; ^6^Pulmonary, Critical Care and Sleep Medicine, Department of Medicine, Baylor College of Medicine, 1709 Dryden Road, Suite 9.70, Houston, TX 77030, USA

## Abstract

*Introduction*. Cigarette smoking contributes to the deaths of more than 400,000 Americans annually. Each day >3,000 children and adolescents become regular smokers. This paper details a new antitobacco educational program titled “AntE Tobacco” *Method*. Children in grades 1–3 were administered a 10-item questionnaire to ascertain their baseline knowledge about the ill effects of smoking, shown an educational cartoon video depicting the ill effects of tobacco, and given a story book based on the video. At the end of video, children were administered a questionnaire to determine short-term recall of the antitobacco educational objectives of the program. Four to 6 weeks later, the children were then administered a follow-up survey to determine long-term retention of the anti tobacco educational program. *Result*. Eighty two percent of the children answered the outcome questions correctly immediately following the video. At follow-up, 4–6 weeks later, 83% of children answered all questions correctly. *Conclusion*. The anti tobacco education program used in this study effectively conveyed most of the educational objectives. The results of this study indicate that a multimedia (i.e., video and book) educational program can be used to educate and reinforce anti tobacco messages. This program may be very useful as a part of a comprehensive anti tobacco curriculum in school systems.

## 1. Introduction

Cigarette smoking contributes to premature deaths of an estimated 443,000 Americans annually, resulting in $193 billion in direct health care expenditures and productivity losses every year [1]. This figure represents more deaths than from AIDS, alcohol abuse, car accidents, murders, suicides, drug usage, and fires combined [2]. For the period of January through June 2008, 20.8% of adults aged 18 years and over were current smokers, which was higher than the 2007 estimates of 19.8%. Over 90% of people that smoke begin before the age of 18, and each day more than 3,000 children and adolescents become regular smokers [3–5]. Studies have shown that the more teens are exposed to cigarette marketing, the more likely they are to smoke [6], and the younger the age at initiation of smoking the greater the risk of habitual smoking [7–9]. In 2006, approximately 6% of middle school students were current cigarette smokers (6% of males and 6% of females) [10]. In addition to cigarette smoking, more than 13% of high school students were current cigar smokers in 2007 (19% males, 8% females) [11]. 

Antismoking programs as part of school curriculum have been shown to be effective in preventing initiation of smoking among children and adolescents [12]. Likewise, evidence suggests that statesponsored antismoking media campaigns may play a potentially effective role in reducing smoking among those exposed to the message [13–18]. In the United States, electronic antitobacco advertising has been used as part of comprehensive tobacco control program in several states and communities [19]. McAlister et al. found that significant reductions in adult tobacco use can be achieved through a combination of intensive media and community campaigns [20]. However, antismoking education has shown little or no effect on students who initiate smoking prior to participation in an antismoking education program [20–24]. In addition, parents' and teachers' opinions and participation may play a role in the success of antismoking programs.

Exposure to smoking in films has been shown to be associated with smoking initiation in young adolescents (aged between 9 and 15 years) both cross sectionally [25, 26] and prospectively [27, 28]. The effect of seeing smoking incidents in films was also stronger in adolescents with nonsmoking parents [25–27]. The tools used to deliver tobacco education may be as important as the message itself, particularly in children. Videos are a popular medium and effective educational tool. They have been shown to be more effective than still pictures or handouts in a variety of learning situations [29]. 

Norum [30] describes how some actions, that are otherwise difficult to illustrate, can be effectively shown to an entire class using video. Most single-component programs [31–34] do not appear to be effective. Thus, using a video education tool in conjunction with a story book is likely to produce greater effects, especially when delivered by health care providers in the school-based setting.

This paper describes a structured antitobacco educational program entitled “*AntE Tobacco*” that was developed using a cartoon story video presentation and an accompanying cartoon storybook that reinforced the educational objectives of the video about the potential harmful effects of smoking. The movie tries to deliver 2 key messages: “say no to smoking the first time and every time” and “stop using tobacco before it kills you.” The cartoon video and storybook were developed under the aegis of the Chest Foundation, the philanthropic arm of the American College of Chest Physicians. The video was watched in a classroom setting, and the storybook was taken home for further discussion with their parents by participants.

## 2. Methods

### 2.1. Permission

Appropriate Institutional Review Board and school board approvals were obtained.

### 2.2. Study Population

The study was conducted as part of an educational program in the Corpus Christi, Callalen, and Flour Bluff school districts of Texas. After receiving approval from school district administrators to conduct an educational program, calls were made to all schools, and programs were conducted on a first come first served basis. Approximately 22 schools in Corpus Christi Independent School District and schools in Callalen and Flour Bluff school districts participated. The study included children from the first, second, and third grades. All the children who were present during the day of presentation participated. The teachers of those students who were present during the presentation were also surveyed.

### 2.3. Methodology

The program was conducted by physicians, physician assistants, medical students, and nurses on a voluntary basis. Each educational team was comprised of 2–4 health professionals as volunteers, and all volunteers underwent a 1-2 hour training session prior to serving as an educator. Each of the volunteers was trained by the master trainer who had done at least 25 lectures on this topic, working from a script, so consistency delivering the lecture was maintained. New volunteers, after the appropriate training (i.e., approximately 1-2 hours of in office training), also observed the delivery of 5 lectures by the trained volunteers and delivery of 5-6 lectures in a supervised setting before being allowed to deliver the lecture independently. Each educational session lasted approximately 45 minutes (to accommodate to school sessions). Each class size was approximately 40–50 students (2 classes were combined most of the times). After introduction to the program by the team leader, all children were administered a 10-item questionnaire (Yes and No) to ascertain their baseline knowledge about the ill effects of smoking (Table 1). The questionnaire evaluated smoking habits of the family (parents, siblings). It also surveyed the children's knowledge of the effects of active and passive smoking, its addictive nature, and their previous smoking prevention education. The lectures, as well as delivery of questionnaires, followed the same format to avoid bias.

**Figure 1:**
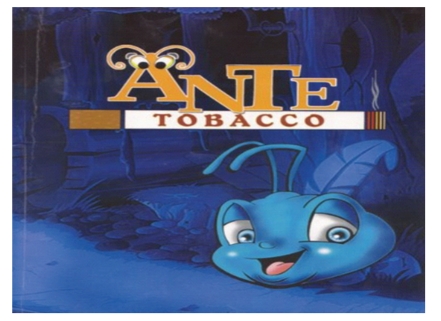


Following the survey of baseline knowledge, the “*AntE Tobacco*” program was viewed. The program consists of a 13-minute educational cartoon video depicting the ill effects of smoking using a family of ants. At the end of the video the children were administered another set of 9 questions (multiple choice) (Table 2) based on the antitobacco cartoon movie to assess retention of the antitobacco messages presented in the movie and storybook. Following administration of the program, all teachers who viewed the program (*n* = 100) completed a survey on their opinions of the usefulness of the program. Following the administration of the program and questionnaire, the children were also given the 40 pages glossy print storybook based on the video to take home for further reinforcement of the educational video (Figure 1). A follow-up visit was done 4–6 weeks later, and students were administered the same questionnaire given postvideo to evaluate their long-term retention.

To assess the children's memory of the video and the impact of “AntE Tobacco” story book, a small group of students were randomly selected to participate in follow-up assessment 4–6 weeks later. The same preset survey was given to the students. Students from all 3 grades (*n* = 679) completed the same set of 9 postvideo questions to assess the effect of the AntE Tobacco program on retaining the antitobacco message.

### 2.4. Data Analysis

The data was analyzed using Students T-test and chi-square test were conducted as part of the Microsoft Excel Data Analysis Package. A *P*-value of <.05 was considered significant. Investigators considered correct response ≤79% as poor, between 80–90% as good, 91–95% as very good, and greater than 95% as excellent responses to questions.

## 3. Results

### 3.1. Students' Component

A total of 6,595 children (1st grade = 2,444, 2nd grade = 2,086, 3rd grade = 2,065) completed the baseline assessment. A total of 6,269 children completed the postmovie questions (1st grade = 2,345, 2nd grade = 1895, and 3rd grade = 2,029) as some students had to leave earlier due to other school assignments, and 1 school's 2nd grade students were excluded as they were inadvertently given a different set of questions. 

The overall baseline knowledge of all grades about the ill effects of smoking, its addictive nature, the effects of passive smoking, and smoking prevention programs was considered good to excellent by the investigators' preset standards. The correct responses for all grades ranged from 81% to 97%. There was a statistically significantly (*P* < .05) higher knowledge level among the 3rd graders compared to 1st and 2nd graders (Table 1).

Approximately 23% of the students' parents and 6% of siblings smoked cigarettes. Over 95% of the children were aware that smoking can adversely affect health. 93% knew that smoking can cause cancer. 87% were aware of the harmful effects of second-hand smoke. Surprisingly, 89% had not been given antitobacco messages. 83% knew that, once started, it was hard to quit the habit. 3% believed they could smoke in school.

Post video, children were administered a set of 9 multiple choice questions to assess short-term memory of the video. The questions asked posttest also assessed retention regarding the intended message as the questions asked were relevant to the educational objective (i.e., the antitobacco education program). Those answers revealed good recall of the video (i.e., poor: < 70, fair: 71–80, good: 81–90, excellent: >91) (Table 2).

To determine the utility of the movie and storybook based on the movie, as an educational tool, 3 schools were randomly selected for a 4–6 week posttest follow-up questions by 1 of the volunteers who did not participate in the educational program. Calls were made to the counsellor or responsible person in those schools to request the 4–6 week follow-up. Two of 3 schools agreed to participate in the 4–6-week follow-up. We surveyed 679 child participants 4–6 weeks after the initial program. Chi square test for 1st, 2nd, and 3rd grades was done separately as shown in Table 3. Compared to baseline, 1st grade students showed a significant improvement in correctly answering 2 of 9 questions (*P* < .05). Second grade students showed a significant improvement in correctly answering 7 of 9 questions (*P* < .05), and 3rd grade students showed a significant improvement in correctly answering 6 of 9 questions (*P* < .05) (Table 3). Most children had the retention level in the range of >90%. The percentage of correct responses to the 4–6 week followup questionnaire by question and grade are summarized in Table 4.

### 3.2. Teachers' Component

We gathered data on the teacher's perception of the video. Ninety eight percent of the teachers liked the program and would like to see it presented again. Ninety seven percent felt that it was age appropriate, and 93% thought that the program will influence the children. In addition, 99% stated that they would recommend it to other classes. Some important suggestions for improvement were to have smaller groups and more time spent with the children. Overall, the teachers considered the *AntE Tobacco *program excellent, useful, and easy to follow by the children. Some of the teacher's comments are as follows:

*“I think this subject needs to be addressed as often as possible."*

*“This program should be presented every year at all elementary schools.”*

*“Great Job!!!!”*

*“The program should be taught to latch key and summer school kids.”*

*“......the cartoon and book are a very age appropriate way to influence and teach the children.”*

## 4. Discussion

A national health objective for 2010 is to reduce the prevalence of current cigarette use among high school students to <16% [35]. Given the current rate of smoking among high school students, this goal may be difficult to achieve unless the downward trend in youth smoking seen between 1997 and 2003 resumes [36]. Jackson et al. [37] reported that early initiators of smoking are at greater risk of becoming habitual smokers if they are exposed to parental and peer modeling of smokers, with lack of parental monitoring and peer pressure against initiating smoking. Therefore, an important step in smoking prevention is to implement tobacco education programs intended to prevent early initiation of smoking.

The current strategies mainly concentrate on middle school prevention programs for adolescents, overlooking the needs of younger children who are at risk for habitual cigarette smoking. However, it is also important that the message is delivered in a way the primary grade children can understand and retain the educational message. This can be a challenging task, as young children have shorter attention spans and may not completely understand the concept of the ill effects of smoking. The popularity and interest in videos among children can be utilized to convey the educational message in such circumstances.

In addition to traditional class room teaching, videos can be used as an excellent tool to expose the minds of children to the ill effects of smoking. The *AntE Tobacco* program was developed based on the belief that a video and cartoon can be used to effectively deliver the antitobacco message. The results of our survey showed that the baseline knowledge about the ill effects of smoking in children was good in the opinion of the investigators. Inadvertently these responses may be somewhat biased as all 10 questions were scaled such that a “yes” answer reflects the desired response that smoking is bad. Children may realize this and simply answer “yes.” Knowledge of the ill effects of smoking increased after the video presentation. The video was effective in reinforcing this knowledge as seen by the responses among all grades immediately following the video. It is likely that using both a video and the cartoon book aided in helping the children retain this message as seen from the responses 4–6 weeks later, as ideally one would have expected to have significant decline in retention about movie at 4–6 weeks.

Our school-based programs conducted by health care professionals using the multiprong approach with discussion, lecture, movie, storytelling, as well as children receiving the story book based on movie to take home as a follow up reading may provide another venue for education.

## 5. Limitations

The current study has limitations. The instruments used in this study have not yet been validated. Since the instruments used were not validated and the study was observational in nature, we cannot make formal statements about power. It was conducted in a specific geographic area (i.e., Corpus Christi, Texas). There was no control group or long-term follow up to evaluate whether this program would lead to a reduction in future smoking among these children. There was no follow up on school that received the program and not the cartoon storybooks (as all children received the take home cartoon storybook). The post video questions follow-up 4-6 weeks later were not paired. We feel that validated questionnaires using the psychometric analysis need to be done to avoid the “yes” bias in the children's understanding of smoking and its hazard. Moreover, well-structured programs need to be designed to assess the impact on future smoking. The socioeconomic status of the family was not correlated with the responses. Nevertheless, we feel this is an important step in assessing the usefulness of implementing anti tobacco educational programs at an early age, and we were able to obtain valuable information from the survey.

## 6. Conclusions

Schools play an important role in shaping student tobacco use behaviors. The popularity of videos among children can be exploited to deliver the antismoking message in an effective way. Antismoking education programs should train the students in refusal skills, involve parents, teachers, and peers in smoking prevention activities, and provide adult role modeling of nonsmoking behavior. The video-based smoking education program used in this study conveyed all of the above very effectively in the form of a cartoon video and a cartoon book based on the video. The results of this study indicate that a multimedia (video and book) educational program can be used to capture the attention of the children, educate, and reinforce retention about the ill effects of smoking. This program may be very useful as a part of a comprehensive antitobacco curriculum in school systems.

##  Financial Disclosure

None of authors has received any financial compensation for this project. D. K. Guntupalli is the creator of AnteTobacco Video. She has not received any monetary compensation or compensation in any other form.

## Figures and Tables

**Table 1:** Percentage of positive responses to prestudy questionnaire by grade.

	Percentage of respondents answering YES			
Question number	All grades *n* = 6595	1st grade *n* = 2444 (37.1%)	2nd grade *n* = 2086 (31.6%)	3rd grade *n* = 2065 (31.3%)

(1) Do your parents smoke?	23	28	25	15
(2) Do your siblings smoke?	6	8	5	3
(3) Is smoking hard to quit?	83	68*	89*	95
(4) Can cigarette smoking hurt you?	93	87*	96*	99
(5) Can cigarette smoking cause cancer?	93	88*	94*	97
(6) Can someone else smoking hurt you?	87	78*	89*	96
(7) Can cigarette start fires?	94	89*	95*	97
(8) Can cigarette smoking make you sick?	96	94*	96*	98
(9) Can anyone smoke in your school?	3	5*	3.5*	1
(10) Have you been told not to smoke?	89	79*	25	98

*P < .05 compared to 3rd graders (Chi square test).

**Table 2:** Percentage of correct responses to poststudy questionnaire by grade.

Question number	All grades (%) *n* = 6457	1st grade (%) *n* = 2345	2nd grade (%) *n* = 2083	3rd grade (%) *n* = 2029

(1) What was Grandpa putting in his mouth?	97	96	100	100
(2) Why was Grandpa walking behind all the ants?	96	95	96	96
(3) What did Tinku and his friend find on the ground?	98	97	99	99
(4) What happened to Tinku and his friend after they ate some of it?	98	97	99	98
(5) What did Grandpa tell the ants?	97	96	98	96
(6) What did Baccy de'ville say to the ants?	79	73	86	78
(7) What did the Fairy say after she reappeared?	97	96	98	98
(8) How did the Fairy help the ants?	97	96	98	95
(9) How did Grandpa feel after Fairy left?	87	86	90	87

**Table 3:** Survey of children 4–6 weeks later using the same set of questions used immediately following the video (same schools).

Questions answered correctly				
	Immediately post	4–6 weeks later	Chi-square	*P* value

1st Grade	*N* = 2345 (%)	*N* = 231(%)		
Question 1	2269 (97)	227 (98)	1.6	.2
Question 2	2227 (95)	220 (95)	0.032	.8
Question 3	2286 (97.5)	226 (98)	0.107	.7
Question 4	2286 (97.5)	226 (98)	0.107	.7
Question 5	2251 (96)	227 (98)	2.9	.08
Question 6	1711 (73)	210 (91)	35	.0001*
Question 7	2251 (96)	219 (95)	0.75	.3
Question 8	2255 (96)	213 (92)	8.1	.004*
Question 9	2016 (86)	210 (91)	4.3	.03

2nd Grade	*N* = 1895 (%)	*N* = 194 (%)		
Question 1	1895 (100)	193 (99)	9.7	.001*
Question 2	1828 (96.5)	178 (92)	10.2	.001*
Question 3	1876 (99)	188 (97)	6.5	.01*
Question 4	1875 (99)	183 (94)	20	.001*
Question 5	1857 (98)	184 (95)	7.7	.005*
Question 6	1629 (86)	178 (92)	5	.02*
Question 7	1857 (98)	186 (96)	3.5	.05*
Question 8	1857 (98)	177 (91)	31	.001*
Question 9	1715 (90.5)	174 (90)	0.13	.7

3rd Grade	*N* = 2029 (%)	*N* = 242 (%)		
Question 1	2029 (100)	238 (98)	34	.001*
Question 2	1960 (96.5)	242 (100)	8.4	.003*
Question 3	2000 (98.6)	241 (99)	1.7	.19
Question 4	1984 (98)	241 (99)	3.5	.06
Question 5	1947 (96)	240 (99)	6.2	.01*
Question 6	1582 (78)	229 (94)	37	.001*
Question 7	1984 (98)	236 (97)	0.06	.7
Question 8	1927 (95)	238 (98)	5.5	.02*
Question 9	1765 (87)	236 (97)	22	.001*

*Statistically significant, *P* ≤ .05

**Table 4:** Percentage of correct responses to the 4–6 week followup questionnaire by grade.

Question number	1st grade (%) *N* = 231	2nd grade (%) *N* = 194	3rd grade (%) *N* = 254
(1) What was Grandpa putting in his mouth?	98	99	98
(2) Why was Grandpa walking behind all the ants?	95	92	100
(3) What did Tinku and his friend find on the ground?	98	97	99
(4) What happened to Tinku and his friend after they ate some of it?	98	94	99
(5) What did Grandpa tell the ants?	98	95	99
(6) What did Baccy de'ville say to the ants?	91	92	94
(7) What did the Fairy say after she reappeared?	95	96	98
(8) How did the Fairy help the ants?	92	91	98
(9) How did Grandpa feel after Fairy left	91	90	97
Overall mean percentage of correct answers	95	94	98
